# Successful diagnosis and management of tuberculosis verrucosa cutis using antituberculosis therapy trial approach

**DOI:** 10.11604/pamj.2020.37.216.26531

**Published:** 2020-11-04

**Authors:** Dina Pebriany, Anis Irawan Anwar, Widyawati Djamaludin, Anni Adriani, Safruddin Amin

**Affiliations:** 1 Dermatology and Venereology Department, Faculty of Medicine, Hasanuddin University, Hasanuddin University Hospital, Makassar, South Sulawesi, Indonesia

**Keywords:** Keywords *: Mycobacterium tuberculosis*, anti-tuberculosis therapy trial, tuberculosis verrucosa cutis

## Abstract

Tuberculosis verrucosa cutis is a paucibacillary form of cutaneous tuberculosis that often occurs in sensitized immunocompetent individuals due to exogenous reinfection. The diagnosis is often difficult because the clinical features are often not typical and acid-fast staining test often shows negative results. Therapeutic trial with antituberculosis therapy is justified if there is strong clinical suspicion in which diagnosis can be made based on the therapeutic response. We report a 46-year-old male with erythematous verrucous plaque on the right knee and crusted erythematous plaque on the left dorsal foot that had been present for 20 years. There were neither history of previous trauma nor tuberculosis treatment. Histopathology, culture, polymerase chain reaction (PCR), Mantoux test, and chest radiograph were negative for cutaneous tuberculosis. Gamma release interferon assay showed positive result. The patient was given category 1 antituberculosis treatment and showed improvement after three weeks. Treatment was continued for 6 months and the lesion significantly regressed.

## Introduction

Tuberculosis (TB) is still a problem in the developed and underdeveloped countries, and cutaneous TB is a small part of extrapulmonary forms. Two most common forms of cutaneous TB are lupus vulgaris and scrofuloderma [1,2]. Tuberculosis verrucosa cutis is a less common paucibacillary form of cutaneous TB caused by exogenous reinfection in sensitized people. Inoculation occurs through wounds or blisters and only rarely from the patient's own sputum [3]. The diagnosis of cutaneous TB is often difficult because the clinical appearance of the lesion is often atypical. Acid fast staining test may be inconclusive in patients with high immune status. Mycobacterial culture is the gold standard for determining cutaneous TB; however, it is often time-consuming. Thus, PCR, which is faster and more sensitive compared to culture, is also often used in clinical practice. Therapeutic trials with antituberculosis therapy are justified if clinical suspicion is strong in which the diagnosis can be made based on the therapeutic response [1,4]. Vora *et al*. stated that even the results of histopathological examination and PCR are often negative especially in paucibacillary cutaneous TB. Antituberculosis treatment trial is sometimes the only solution [5]. Belgaumkar *et al*. stated that in cases of strong clinical suspicion but doubtful laboratory tests, dramatic response to antituberculosis therapy after 4 weeks can be considered a valuable diagnostic criteria [6]. The report discusses a 46-year-old patient who was diagnosed as tuberculosis verrucosa cutis and given a trial of category 1 antituberculosis therapy, who subsequently showed significant clinical improvement after three weeks.

## Patient and observation

**Figure 1:**
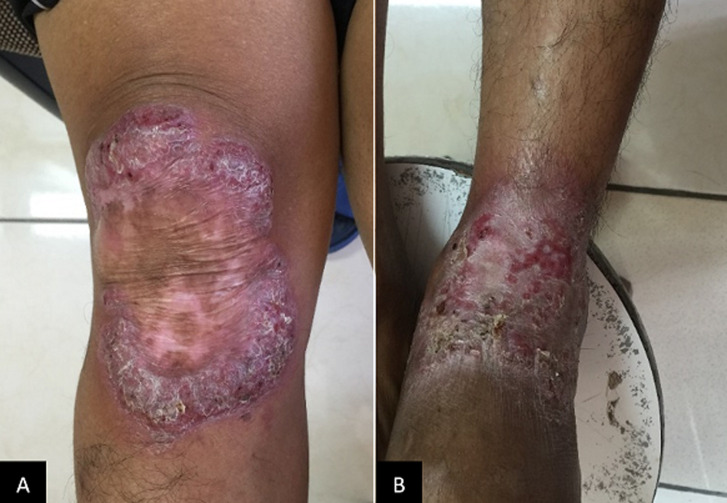
clinical appearance at the first visit. Left knee region shows reddish plaque with verrucous surface, elevated edge and erythematous case with accompanied by crusting. Left dorsum pedis showed reddish plaque and patch with crusting

A 46-year-old fisherman came to dermatovenereology outpatient clinic with a chief complaint of a raised red-purple rough spot on the right knee as well as reddish patches on the left foot for almost 20 years before admission. The lesion initially appeared on the right knee as small and soft lumps which subsequently elevated and grew larger in size. Moreover, the lesion easily bled upon minor trauma and eventually produced pus mixed with blood. The patient sometimes complained of itching but no pain was reported. The patient denied a history of long-standing cough, bloody cough, night sweats, weight loss, and there was no history of TB treatment. Patient also denied any histories of allergy to food or drugs as well as history of family with the same complain. History of immunization was unknown. The patient had been treated at the local hospital in multiple occasions with both oral medication and ointments, but the complaints did not show any improvement. The patient was in an otherwise healthy condition with a body weight of 64 kg. Dermatological examination of the right knee showed erythematous plaque with verrucous surface, elevated edge and erythematous base accompanied by crusting. Left dorsum pedis showed erythematous macules and plaques accompanied by crusting (Figure 1). The differential diagnosis of this patient included tuberculosis verrucosa cutis, lupus vulgaris, chromoblastomycosis, and neurodermatitis, with tuberculosis verrucosa cutis as the working diagnosis.

**Figure 2:**
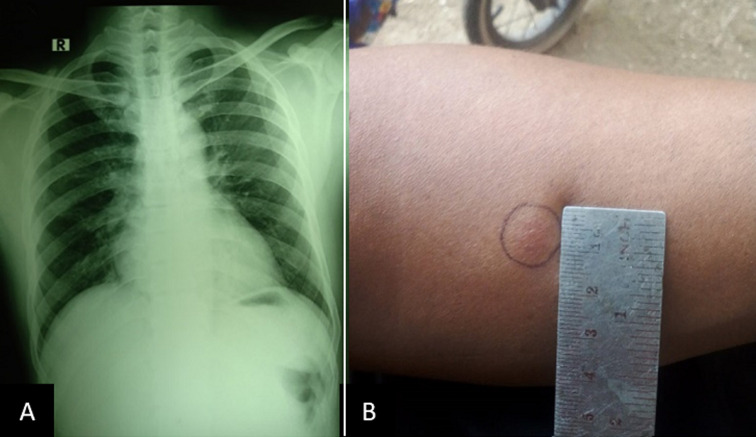
chest radiograph and Mantoux test: (A) normal result of chest radiograph; (B) negative result of Mantoux test

**Figure 3:**
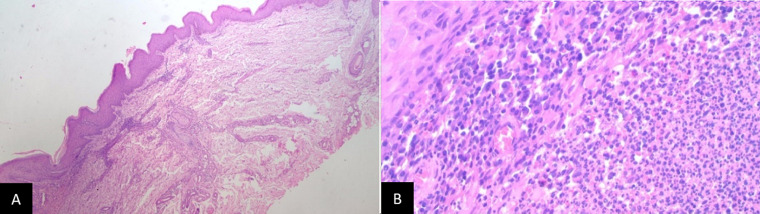
histopathological result showed (A) signs of inflammation in dermis (4x magnification) and (B) lymphocyte and neutrophil (40x magnification)

Laboratory test showed leukocytosis (11,350/uL) while other results of complete blood test, blood sugar, and liver functions were within normal range. Examination of pus and skin scraping by Ziehl Nielsen staining did not reveal any acid-resistant bacilli (AFB). Skin scraping examination with 10% KOH revealed no hyphae, spores nor muriform bodies. There was no visible fungus growth in tissue culture with saboraud agar. Chest radiograph did not show any abnormalities (Figure 2A). Mantoux test showed negative result (induration of 9 mm) (Figure 2B). Histopathological examination of skin tissue from the left knee showed pseudoepitheliomatous hyperplasia of the epidermis and diffuse lymphocytic and neutrophil inflammation infiltrate in the superficial dermis extending to the epidermis, perivascular areas, and the interstitial middle and deep dermis. Periodic acid Schiff (PAS) staining did not show presence of fungal elements. The final conclusion of the histopathological examination was suppurative chronic inflammation which can be found in deep mycosis and cutaneous TB (Figure 3A, B). Due to this inconclusive result, further tests in the form of *M. tuberculosis* culture with skin tissue sample, Interferon-gamma release assays (IGRA) test with ELISA method using blood sample, and examination of *M. tuberculosis* (MTb) with GeneXpert method using tissue sample, were carried out. MTb examination using GeneXpert method and culture after four weeks failed to demonstrate the presence and growth of *M. tuberculosis*, respectively. However, the result of IGRA examination was positive.

**Figure 4:**
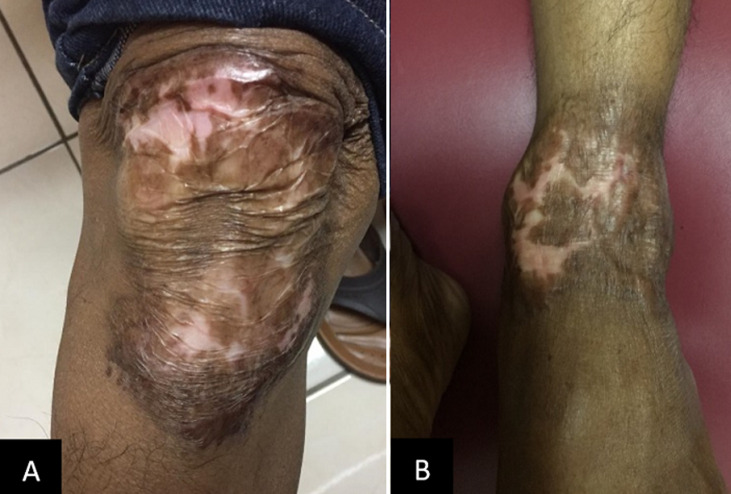
significant lesion improvement was evident after six months

Based on the history taking, physical examination, and supporting tests, the patient was diagnosed with tuberculosis verrucosa cutis and given category 1 antituberculosis therapy trial. Based on the 64 kg body weight of the patient, four tablets of fixed dose combination antituberculosis drugs each consisted 150 mg rifampicin, 75mg isoniazid, 275 mg etambuthol, and 400 mg pyrazinamide were given once daily for 4 weeks. In addition, B Complex tablet was also prescribed once daily. After 21 days of treatment, thinning of the plaque and significant improvement of the pruritus were observed. In addition, there were no adverse reactions reported by the patient. Therefore, the regimen was continued for two months before being converted to the maintenance phase in the third month. In the maintenance phase, two tablets of fixed combination drugs each consisted of 75 mg isoniazid dan 150 mg rifampicin were given. At the end of the six-month treatment, a significant improvement was evident (Figure 4).

## Discussion

The diagnosis of cutaneous TB in this case was established through history taking, clinical appearances, and good clinical response to the use of antituberculosis therapy. High-risk groups for this disease include doctors, pathologists, laboratory workers, farmers, butchers and veterinarians with the prevalence of male is higher than women (2: 1). Tuberculosis verrucosa cutis often occurs exogenously after *M. tuberculosis* inoculation into the skin and appears as a growth of purplish-colored mass or brownish red warts which becomes hyperkeratotic and resembles warts (warty papules) with an erythematosus base. Lesion is usually without pain and systemic symptoms with predilection sites on the knees, elbow joints, hands, feet and buttocks. Lesions are often seen on the legs of the pediatric population, while the involvements of arm are more common in adults [2,5,6]. Tuberculosis verrucosa cutis has a slow progressive course. The lesions are initially in the form of deep-seated papules or nodules which gradually enlarge and became verrucous. Sometimes purulent discharge and crusting can be observed. Lesions are usually single with irregular peripheral extensions, hence producing central healing with atrophic scars. Firm and enlarged regional lymph node can be found upon regional examination [7].

The source of tuberculosis verrucosa cutis infection in this patient was thought to originate from autoinoculation that occurred through a small wound that was initially not recognized by the patient. In light of TB infection which most often infects the lungs, it is thus deemed necessary for any patients suspected with tuberculosis verrucosa cutis to undergo chest X-ray to look any evidence of focal infection in the lungs. However, the result of chest radiograph in the current patient was normal and the result of bacteriological examination from skin scraping and pus with Ziehl Nielsen's staining showed no acid-fast bacilli. This situation is in accordance with the literature which reports that acid-fast bacilli are not always found in tuberculosis verrucosa cutis due to the paucibacillary nature of tuberculosis verrucosa cutis [3]. Histopathologic examination from the lesion on the right knee showed suppurative chronic inflammation which was not specific for cutaneous TB as it could also be found in deep fungal infections. PAS staining showed no fungal element. Histopathological features of cutaneous TB are characterized by epidermal pseudoepitheliomatous hyperplasia with hyperkeratosis and inflammatory infiltrate cells consisting of neutrophils, lymphocytes, and giant cells. Cardinal sign of cutaneous TB is the presence of granulomatous infiltrates. The typical TB focus with caseosa necrosis is rarely seen in tuberculosis verrucosa cutis [8].

Due to the inconclusive result, further tests were carried out in the form of *M. tuberculosis* culture from the skin tissue, IGRA test with ELISA method, and MTb examination with GeneXpert method, which all showed negative result except for the IGRA test. According to Gopinathan *et al*. in certain circumstances only 50% cases showed growth of *M. tuberculosis*, presumably because some cases of skin tuberculosis were paucibacillary infection [9]. Belgaumkar *et al*. reported that GeneXpert MTB/RIF test based on PCR yielded in a rapid result and high sensitivity in patients without pulmonary TB infection, especially those with HIV coinfection. However, although this result made it a potential tool for extra-pulmonary specimens such as the skin, there are not many reports yet showing its use [6]. The positive IGRA result led to the possibility that the patient was infected with *M. tuberculosis*. IGRA is a serological test that assesses latent infection by measuring interferon-gamma produced by T-cells in individuals exposed to the Mtb antigen. There are two tests approved by the FDA, the QuantiFERON-TB Gold (QFT-G) and T-SPOT [10]. The most possible differential diagnosis of this case was chromoblastomycosis which is a deep fungal infection with a similar clinical presentation. The diagnosis is established by finding muriform body on potassium hydroxide (KOH) or histopathological examination [2]. To rule out chromoblastomycosis, 10% KOH examination and fungal culture with saboraud media should be performed and showed no hyphae, spores nor muriform bodies and culture test showed no fungal growth.

In the state of paucibacillary types, such as tuberculosis verrucosa cutis, culture sensitivity is low. In addition, culture takes weeks to complete and hence may lead to delayed treatment. In some studies, microscopic, culture, and PCR are often negative. Tan *et al*. found that PCR can provide 100% sensitivity and specificity in multibacillary TB but in cases of paucibacillary TB, the positive rate was only 55% in tuberculosis verrucosa cutis, 60% in lupus vulgaris, and 73% in all cases of cutaneous TB [3,10] Hjira *et al*. reported that therapeutic trials with antituberculosis therapy are justified if clinical suspicion is strong where diagnosis can be made based on therapeutic response.[1] Vora *et al*. stated that even histopathological examination and PCR can also complicate the establishment of diagnosis due to the paucibacillary nature of the disease. Thus, sometimes treatment trials with antituberculosis therapy are the only solution [5] Belgaumkar *et al*. also stated that in cases with strong clinical suspicion but doubtful laboratory tests, dramatic response to antituberculosis therapy after 4 weeks is considered a valuable diagnostic criteria [6]. We decided to administer a trial of antituberculosis therapy for four weeks in this case which showed a significant improvement after 21 days. No side effect was reported and thus the treatment was continued for 6 months. At the end of the treatment, significant improvement was evident as shown by the lesion regression and resulting atrophic scar. Treatment of cutaneous TB is the same as treatment in other organs, in which category 1 antituberculosis therapy serves as the first option. Treatment of TB is divided into two stages: the initial (intensive) and maintenance stages. In the initial phase, a combination of 600 mg rifampicin, 300 mg isoniazid, 1600 mg pyrazinamide, and 1300 mg ethambutol were given every day for two months. Following this phase, a maintenance phase which consisted of 600 mg rifampicin and 300 mg isoniazid were given three times weekly in the next four months. Second line drugs are streptomycin, cycloserine, levofloxacin, moxifloxacin, gatifloxacin, amikacin, kanamycin capreomycin, clofazimine which are given to patients unresponsive to the first-line treatment or to HIV patients who are infected and have multidrug resistant (MDR) [9,10].

## Conclusion

Cutaneous TB may present with varying clinical manifestations which leads to diagnostic conundrum. In such cases, therapeutic trial using antituberculosis therapy may be justified and diagnosis can be established by assessing the clinical response.
